# Influence of forest structural complexity on small mammal body condition and its impact on tick burden and pathogen prevalence

**DOI:** 10.1186/s13071-025-06874-0

**Published:** 2025-06-18

**Authors:** Tosca Vanroy, Lander Baeten, An Martel, Bram Catfolis, Manoj Fonville, Luc Lens, Frank Pasmans, Hein Sprong, Diederik Strubbe, Elin Verbrugghe, Kris Verheyen

**Affiliations:** 1 Forest & Nature Lab, Department of Environment, Faculty of Bioscience Engineering, Ghent University, B-9090 Melle-Gontrode, Belgium; 2 Wildlife Health Ghent, Department of Pathobiology, Pharmacology and Zoological Medicine, Faculty of Veterinary Medicine, Ghent University, B-9820 Merelbeke, Belgium; 3 Terrestrial Ecology Unit, Department of Biology, Faculty of Sciences, Ghent University, B-9000 Ghent, Belgium; 4 National Institute for Public Health and the Environment (RIVM), Bilthoven, the Netherlands

**Keywords:** *Apodemus sylvaticus*, *Myodes glareolus*, Tick-borne pathogens, Scaled mass index, Telomere length

## Abstract

**Background:**

More and more forest management focuses on increasing structural complexity to improve environmental conditions for biodiversity and forest functioning. However, it remains uncertain whether animal populations also benefit from increased forest structure. Small mammals are key reservoirs for zoonotic diseases, so understanding how forest structure changes their condition and how this, in turn, affects infection dynamics is critical for animal and human health.

**Methods:**

This study examined relationships between forest structural complexity, individual body condition (scaled mass index (SMI) and telomere length), pathogen prevalence, and tick load in bank voles and wood mice across 19 forest plots in northern Belgium, representing a gradient of structural complexity.

**Results:**

Results showed that higher forest complexity, especially with more dead wood and a well-developed herb layer, increased small mammal abundance. Density varied by tree species, with highest abundances in oak and lowest in poplar forests. In addition, body condition improved with structural complexity; SMI increased with woody layer complexity in wood mice and with dead wood availability in bank voles. No clear relationship between telomere length and forest complexity was observed. The relationship between body condition and pathogen prevalence was species- and pathogen-specific. Small mammals in better body condition were more likely to host *Borrelia burgdorferi* (causing Lyme disease), particularly in complex forests, indicating a higher infection risk with increasing structural complexity.

**Conclusions:**

Forest management practices that aim to enhance forest structure and biodiversity may thus inadvertently increase zoonotic disease risk and should take these findings in consideration to minimize the risk for human health.

**Graphical abstract:**

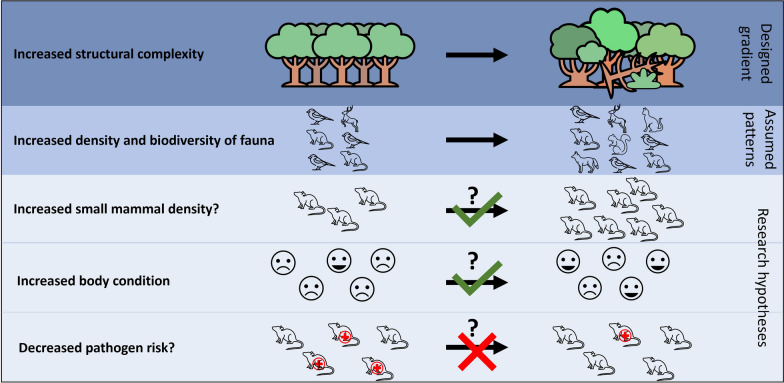

**Supplementary Information:**

The online version contains supplementary material available at 10.1186/s13071-025-06874-0.

## Background

Humans have been impacting forests for a long time, their efforts often aimed at maximizing wood production through the conversion of natural forests into structurally simple, fast-growing monocultures. Recently, however, there has been a shift toward forest management strategies that prioritize forest resilience, biodiversity, and multifunctionality [1–4]. This shift includes increasing the structural complexity of forests, which can be achieved by enhancing both the horizontal and vertical structure through increasing tree species diversity, the accumulation of dead wood, and the increased cover of herb and shrub layers [2, 5]. Increasing forest structural complexity creates a more heterogeneous habitat, expanding the multidimensional niche space. This expansion facilitates the coexistence of a greater number of species and leads to an increase in their abundance, at least in temperate forests [6], not only in the plant community but also in the wildlife that inhabits these forests.

The abundance of small mammals is influenced by forest structural complexity, in particular by the presence of dead wood and shrub cover, which create vital microhabitats [7–10]. Rodents, in particular, are important components of forest ecosystems, functioning as seed dispersers, predators, and preys [11], and their abundance is often used as an indicator of habitat quality [6, 12]. Relying simply on rodent abundance as an indicator of habitat quality, however, can be misleading, as high densities may persist in low-quality habitats due to immigration, and low densities might be found in high-quality habitats due to disease predation or seasonality of habitat [8, 13, 14]. Focusing on individual-based pathways might give us a better idea of the physical condition of an individual.

Higher population densities do not necessarily reflect better physical condition of individuals. Competition might increase with density, which could negatively impact food availability per individual or increase stress between individuals, having a negative effect on their condition. A reliable indicator for the physical condition of an individual is body condition, which is commonly used in ecological studies [15, 16]. Body condition reflects an animal’s energy reserves, accounting for the balance between energy gained from feeding and energy expended on survival and reproduction. Key factors influencing body condition include food availability and protection from predation [17]. Understory vegetation and dead wood can provide food such as seeds, fruits, and insects, but they can also provide cover against predators [10, 12, 18, 19]. Therefore, we expect that increased forest structural complexity, particularly a richer herbal layer and greater amounts of dead wood, will enhance the body condition of small mammals.

The body condition of individual rodents can in turn influence their susceptibility to pathogen infections. In general, it is assumed that individuals in poorer condition are more susceptible to pathogens. Animals with a better body condition, and thus more energy reserve, can use this energy for their immune defense. When food availability is low, individuals will have to make a trade-off between using energy for immune defense or to store the energy to protect them from starvation [20]. Chronic stress can lead to a less effective immune response [21, 22]. Sanchez et al. [23] conducted a meta-analysis that showed an overall negative relationship between body condition and infection. Understanding the relationship between body condition and pathogen prevalence is important in untangling the effects of forest management on zoonotic disease dynamics in wildlife populations [24, 25].

Wild mammals, especially rodents, play a crucial role in many infectious disease cycles, of which some are shared with humans [26–28]. Bank voles (*Myodes glareolus*) and wood mice (*Apodemus sylvaticus*) are reservoirs for several tick-borne pathogens and important feeding hosts for larvae and nymphs of *Ixodes ricinus* ticks [29–32]. These rodents are, therefore, an ideal model for studying disease ecology owing to their potentially high densities, their role as reservoir hosts for human pathogens, and their importance as feeding hosts for ticks [32]. For humans, the risk of contracting a tick-borne pathogen depends on both the density of ticks and the infection prevalence within the tick population. Since ticks acquire their infection from infected hosts, the tick’s infection prevalence is directly linked to the infection prevalence of the reservoir hosts. Consequently, changes in rodent population densities can influence disease risk. According to the amplification hypothesis, an increase in host abundance is likely to lead to an increase in pathogen prevalence [33, 34]. Poorer body condition among reservoir hosts may furthermore enhance their susceptibility to infection, thereby increasing the likelihood of zoonotic disease transmission to humans [35]. Alternatively, the dilution hypothesis assumes that when host diversity increases, the proportion of incompetent hosts (for a given pathogen) will also increase and the prevalence of tick-borne pathogens will decrease [36].

Tick-borne pathogens represent a growing public health concern worldwide [37]. As primary vectors for a diverse array of viruses, bacteria, and parasites, ticks transmit several zoonotic diseases. Among these, Lyme borreliosis stands as the most prevalent tick-borne illness in Europe, caused by spirochete bacteria within the *Borrelia burgdorferi* sensu lato (s.l.) complex [38]. This complex encompasses multiple pathogenic species, including *Borrelia afzelii*, *B. bavariensis*, *B. burgdorferi* sensu stricto, *B. garinii*, *B. spielmanii*, and *B. valaisiana*, each contributing to the epidemiological burden of Lyme disease across the region. On average, almost 130,000 cases of Lyme borreliosis are reported in Europe per year [39]. Other bacteria, such as *Anaplasma phagocytophilum*, can also cause diseases in humans, though their symptoms are often nonspecific and relatively mild [40, 41]. *Spiroplasma ixodetis* and *Rickettsia helvetica* can also cause disease in humans, but these are more occasional human pathogens [42]. All pathogens are known to occur in bank voles and wood mice; however, both species are only reservoirs for *A. phagocytophilum*, *B. afzelii*, *B. bavariensis*, *B. spielmanii*, *B. burgdorferi* sensu stricto, *S. ixodetes*, and *R. helvetica* [40, 43–48].

In this study, we utilized a network of forest plots with a gradient of forest structural complexity replicated with three dominant tree species—pedunculate oak (*Quercus robur*), European beech (*Fagus sylvatica*), and hybrid poplar (*Populus* × *canadensis*). Our objective was to investigate the relationship between forest structural complexity, small mammal density, individual condition, and the infection prevalence in small mammals. First, we studied the effect of increasing forest structural complexity (as explained in Catfolis et al., 2023), its four subindices, and dominant tree species on the abundance of small mammals, focusing on two prevalent species: wood mice (*A. sylvaticus*) and bank voles (*M. glareolus*). Next, we assessed the effect of forest structural complexity, its four subindices, and dominant tree species on two body condition variables (scaled mass index (SMI) and telomere length). Finally, we explored how these two body condition variables influenced the likelihood of pathogen infection (*A. phagocytophilum*, *B. burgdorferi* s.l., *R. helvetica*, and *S. ixodetes*) and the tick burden. We expected to find an increase in the number of captured individuals with higher forest structural complexity, that individuals in more structurally complex forests would have better body condition, and that individuals in better body condition would have a lower probability of infection with pathogens and have a lower tick burden.

## Methods

### Study area

**Fig. 1:**
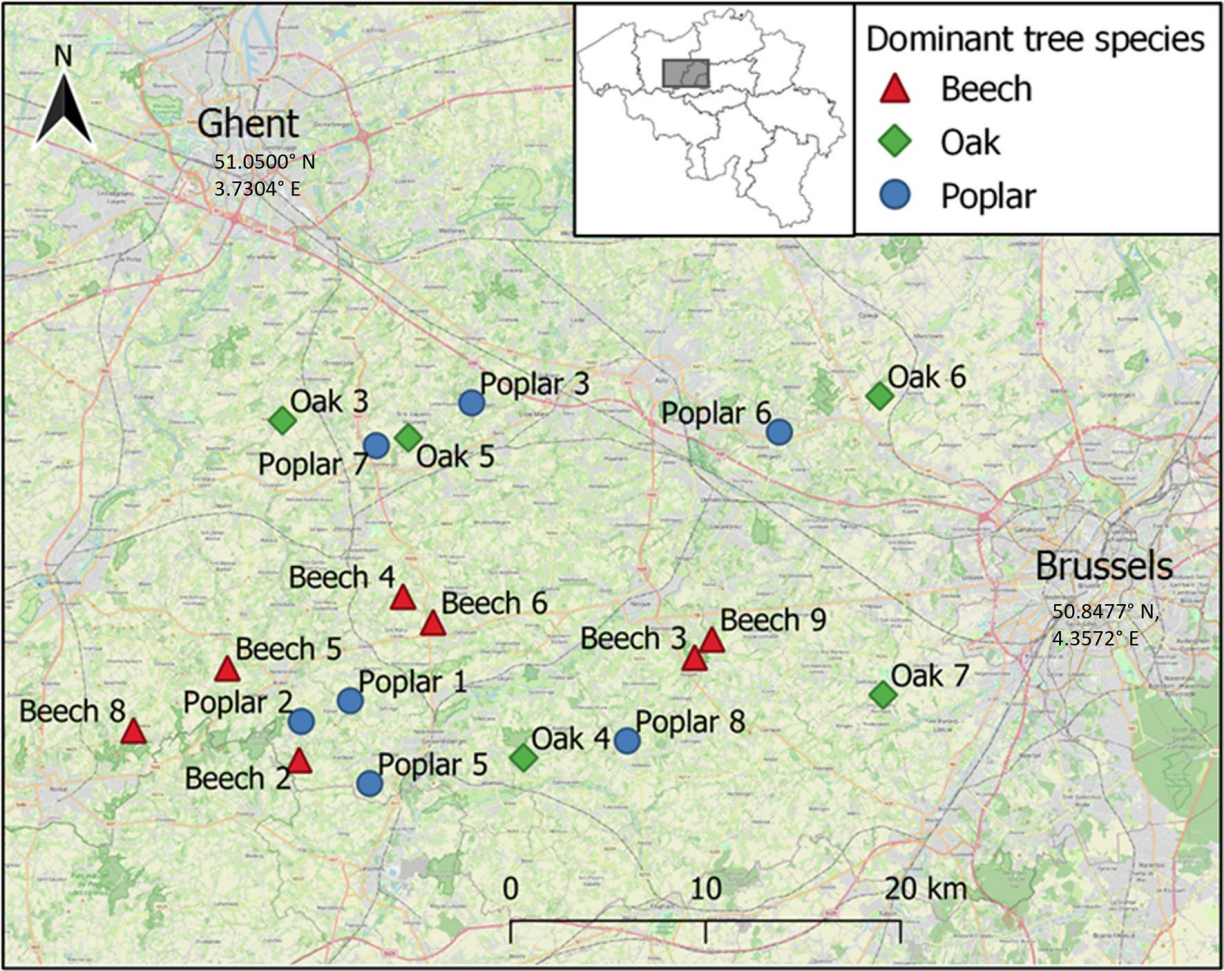
Map of the study sites in Flanders (Belgium). The colored symbols show the dominant tree species of that forest

In a region in Belgium, called the Flemish Ardennes and the Pajottenland, we selected 19 forests for this study to cover a gradient in forest structural complexity (Fig. 1). Variation in other variables such as potential natural vegetation (oak–beech forest), history (forest since at least 1850), and area (at least 3 hectares (ha)) was minimized [50, 51]. Plots were located at least 1 km apart from each other. These forests were categorized into three groups, according to their dominant tree species: oak (*Quercus robur*), beech (*Fagus sylvaticus*), and poplar (*Populus* × *canadensis*).

### Forest structural complexity

A 1-ha “core plot” was established in each forest, where all sampling activities were conducted. Forest structure characterization followed the protocol outlined in the Flemish Forest Inventory [52]. An estimation was made of the degree of the canopy cover, number of stories, stand age, and spatial tree species mixture for the whole 1-ha core plot (homogeneous or heterogeneous). Tree circumference was recorded at breast height (1.3 m), and the length of lying dead wood was measured using the line intersect method [53]. The percentage cover of the herbal, shrub, and tree layer was calculated for each species in a 10 m × 10 m plot in the center of the 1-ha core plot. These data were then used to calculate the Structural Complexity Index (SCI), as described by Catfolis et al. [49] (Additional File 1: S1). The SCI ranges from 0 to 1, a low score (closer to zero) indicating a simpler forest structure and a high score (closer to 1) signifying a more complex structure. The SCI is composed of four uncorrelated sub-indices, i.e., forest structure (vertical and horizontal stand structure), woody layer (species composition and diameter of the threes), herb layer (species composition of the herbal layer), and dead wood (standing and lying dead wood; Table 1).

**Table 1:** Overview of the four sub-indices and their composing variables *DBH* diameter at breast height

Forest structure	Woody layer	Herb layer	Dead wood
Canopy cover	No. of tree species	No. of plant species	Basal area standing dead wood (m^2^/ha)
Stand age (years)	Number of large trees (DBH > 40 cm)	Degree of rareness	Standard deviation in diameter dead wood
No. of stories	No. of very large trees (DBH > 80 cm)	Total cover (%)	Large dead trees
Spatial tree species mixture	Natural regeneration		Total length of lying dead wood (m)
	Standard deviation of DBH (cm)		Number of dead wood classes

### Small mammal sampling and pathogen detection

Small mammals were captured during August and September 2021 using live traps (Trip Trap) baited with oats, peanut butter, raisins, and mealworms. Trapping took place in a grid of 7 × 7 traps with 5 m spacing between traps. Traps were placed the night before and pre-baited. On the night of the trapping, traps were activated 1 h before sundown and checked twice, with intervals of 3 h, before being closed. Small mammals were captured for one night per plot. Each captured animal was identified to species and sex, and measurements of body mass and head–body length were taken. When possible, urine and feces were collected. Each individual was checked for ticks, with special attention to the head and abdomen; any ticks present were removed and stored in 70% ethanol. From each animal, a small piece of ear tissue was collected using an ear punch (VWR, 2 mm, plier-style) and stored at −80 °C. DNA extraction from these ear tissue samples was performed using the Qiagen DNeasy Blood and Tissue Kit (Qiagen, Hilden, Germany).

A total of 11 pathogens were considered: *Anaplasma phagocytophilum*; *Babesia microti*; *Borrelia miyamotoi*; *B. afzelii*; *B. bavariensis*; *B. burgdorferi* sensu stricto*; B. garinii*; *B. spielmanii*; *B. valaisiana*; *Spiroplasma ixodetis*; and *Rickettsia helvetica*. *B. burgdorferi* sensu lato (s.l.) complex consists of *B. afzelii*, *B. bavariensis*, *B. burgdorferi* sensu stricto, *B. garinii*, *B. spielmanii*, and *B. valaisiana*. Pathogen detection for *Anaplasma phagocytophilum* [54], *B. burgdorferi* s.l. [55], *Rickettsia helvetica* [56], and *Spiroplasma ixodetis* [57] was carried out using quantitative polymerase chain reaction (qPCR) on the ear tissues of the small mammals. Small mammal trapping was performed with permission of the land owner and the Flemish authorities (Agentschap voor Natuur en Bos; ANB/BL-FF/V21–00034). All sampling protocols used were approved by the Ethical Committee VIB Ghent site (EC2020–095).

### Statistical analysis

First, we calculated the SMI and the telomere length for each individual. SMI is a proxy for body condition whereby the body mass is scaled for an average body size [16]. First, we regressed log-body mass against log-body length using a linear model. The regression slope for *A. sylvaticus* was 1.80 and for *M. glareolus* 1.33, with an average head–body length of 7.6 cm and 8.1 cm, respectively. SMI was calculated per individual as body mass × (average head–body length/head–body length) ^slope^. Telomeres, which are repetitive nucleotide sequences at the ends of chromosomes, protect the genome during cell division. They become shorter with each cell division due to aging and exposure to stress [24, 58]. Telomere length is often used as a proxy for age and fitness [24, 59, 60]. Relative telomere length was measured using quantitative PCR (qPCR), following the method described by [61], which uses the ratio between telomere repeat copy number versus a single-copy number control gene relative to a reference sample (Additional File 1: S2).

To analyze the effect of SCI on the number of captured individuals, generalized linear models were constructed using the brms package [62] in R 4.4.0 [63]. The total number of small mammals was first regressed against forest area (log normalized), the four SCI sub-indices (Table 1), and dominant tree species as explanatory variables. Then, we simplified the model, replacing the four sub-indices by the overall SCI index. The two models were also fitted for either the number of *A*. *sylvaticus* or *M*. *glareolus* separately to explore differences between species. All models assumed the count data to follow a Poisson distribution. For all analyses, a credible interval of 68% and 90% was used.

Next, the effect of SCI on each of the body condition variables (SMI and telomere length) was evaluated for each species separately, to account for their different ecology. No collinearity was found between SMI and telomere length. For each species and body condition parameter, two generalized linear mixed models were performed. The first model included the four SCI sub-indices, forest area, and dominant tree species as explanatory variables. Forest area was included as a covariate because it is a characteristic of the forest at a local scale that can potentially influence the populations, and it was not included in the structural complexity index. As multiple individuals were measured in the same plot, plot ID was added as a group-level (random) effect. The second model replaced the four sub-indices again with the overall SCI score. All models assumed the data to follow a Gaussian distribution.

Finally, the relationship between body condition parameters and either the pathogen prevalence or tick numbers (or presence) was assessed. The explanatory variables included SMI and telomere length and plot ID as group-level (random) effect. The presence/absence of at least one tick-borne pathogen, *Anaplasma phagocytophilum*, *Rickettsia helvetica*, *B. burgdorferi* s.l., and *Spiroplasma ixodetis*, and the number of ticks or presence of ticks was used as the response variable in separate models. A total of 11 regression models were performed: 6 for *M. glareolus* and 5 for *A. sylvaticus* (the sample size for *Anaplasma phagocytophilum* in *A. sylvaticus* was too low to model). The models using presence/absence data as a response were logistic regressions. The models with the number of ticks assumed the data to follow a Poisson distribution.

## Results

### Effect of forest structural complexity on the number of *A. sylvaticus* and *M. glareolus*

**Fig. 2:**
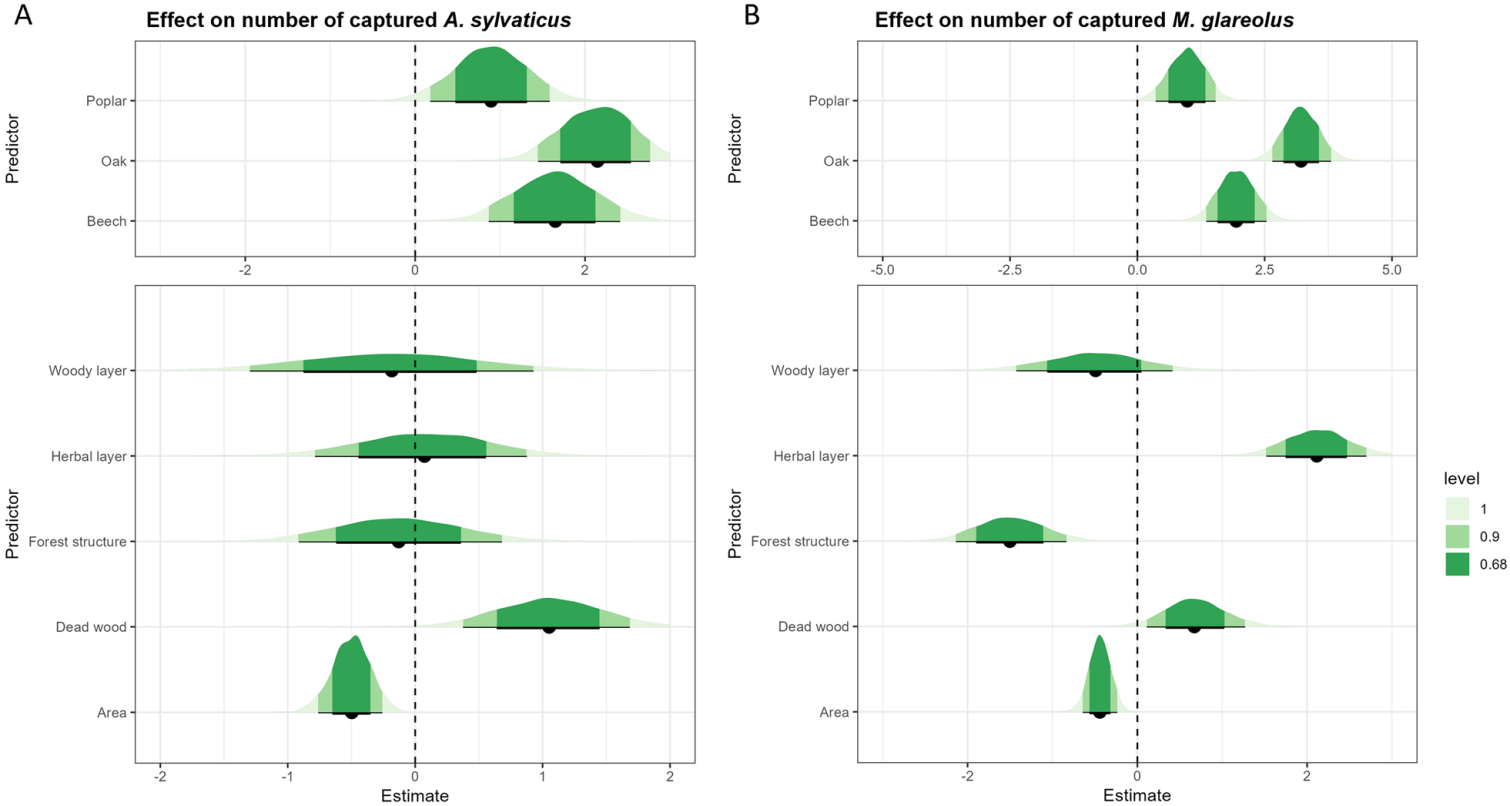
The effect of forest type (top panel), the four sub-indices of the structural complexity index and forest area (bottom panel) on the number of captured (**A**)* Apodemus sylvaticus* and (**B**)* Myodes glareolus*. Top panel: The estimated number of individuals captured for each dominant tree species; a value of two represents for instance 2 individuals/ 900 m^2^. Estimates are for an average-sized forest (scaled area = 0) with complexity equal to zero. Bottom panel: The estimated effects of the four sub-indices of the structural complexity index and forest area on the number of captured individuals of each species; a value of one now represents an increase in the number of individuals when the predictor increases by one unit. The credible interval is shown for 100% (light green), 90% (green) and 68% (dark green). Estimates in the top and bottom panel were drawn from the same statistical regression model (see methods) but simply displayed on different scales for clarity

In total, 314 small mammals were captured across the 19 forest plots, comprising 123 *A. sylvaticus* and 187 *M. glareolus*. In addition, two *Microtus subterraneus*, one *Sorex araneus*, and one *S. minutus* were captured but excluded from the analysis owing to their low numbers. Our data showed strong evidence (95% credible interval (CI) not containing zero) for a negative effect of forest area on the number of individuals captured for both species as well as for the two species combined (Fig. 2 and Additional File 1: Supplementary Fig. S2). The estimated number of individuals, for a forest of average area and a SCI of zero, was highest in oak forests, followed by beech, and lowest in poplar (Fig. 2 and Additional File 1: Supplementary Fig. S2a). No statistically clear effect of the overall SCI was found (Additional File 1: Supplementary Fig. S3a, b). Parameter estimates and 95% credible intervals are presented in Supplementary Table S3 for all models.

When assessing the effect of the four sub-indices of structural complexity, our data suggest strong evidence (95% CI not containing zero) for a positive effect of the score of dead wood on the number of *A. sylvaticus* captured (Fig. 2a). For the number of captured *M. glareolus*, we found strong evidence (95% CI not containing zero) for a negative effect of the forest structure score and weak evidence (90% CI) for a positive effect of the herbal layer score and dead wood score (Fig. 2b).

### Effect of forest structural complexity on individual SMI and telomere length

Owing to incomplete measurements of body mass, length, or ear punctures, data from 120 *A. sylvaticus* and 169 *M. glareolus* were used in subsequent analyses. The mean SMI, calculated for each species separately, was 17.87 (± 3.23) for *A. sylvaticus* and 17.70 (± 2.33) for *M. glareolus*. Mean telomere length was overall shorter for *M. glareolus* (−0.53 ± 0.24) than for *A. sylvaticus* (0.65 ± 0.26).

**Fig. 3:**
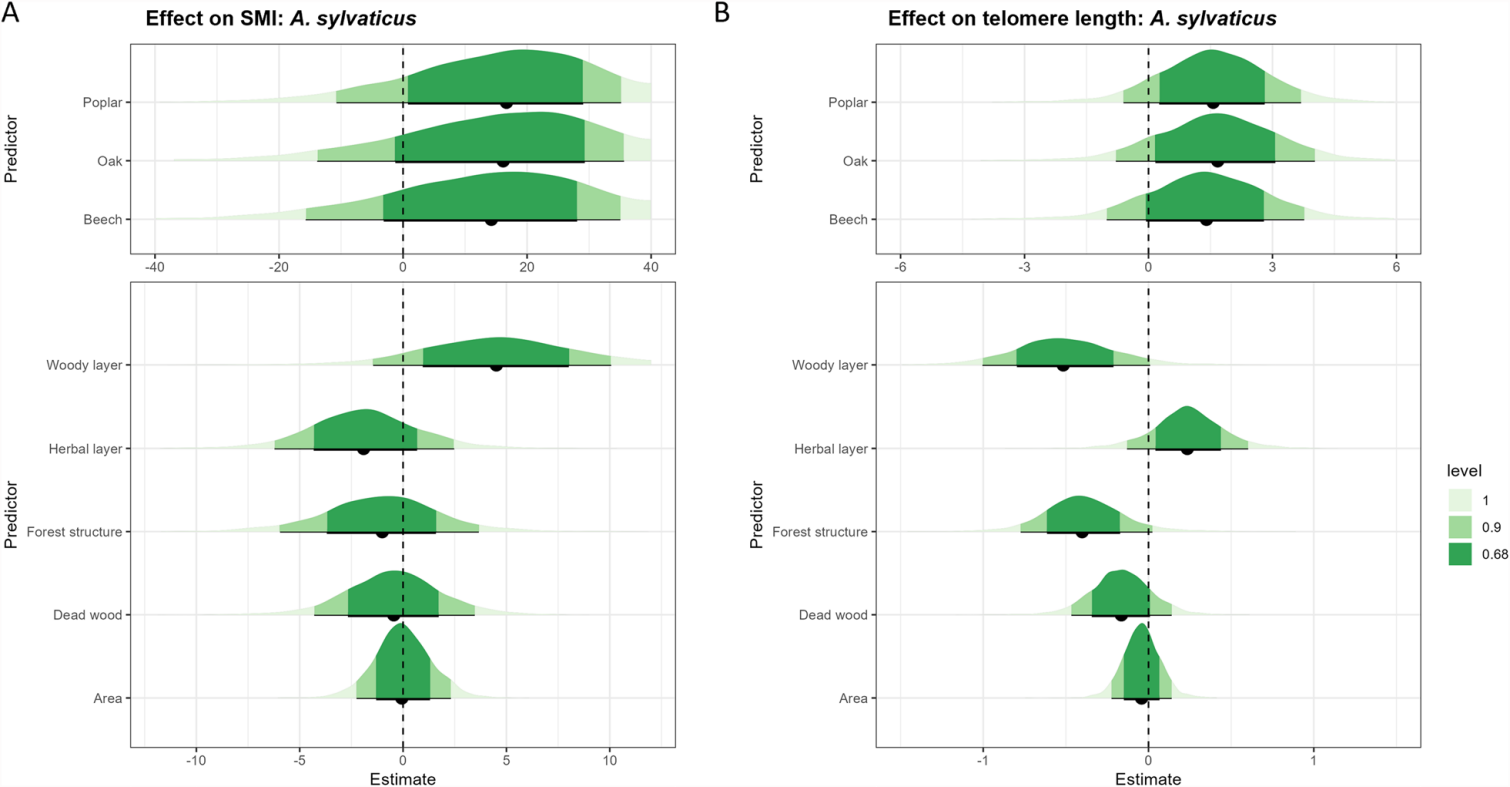
The effect of forest type (top panel); the four sub-indices of the structural complexity index and forest area (bottom panel) on individual body condition parameters in *Apodemus sylvaticus*: **A** scaled mass index and **B** telomere length. Fig. 2 caption provides interpretation guidance. The credible interval is shown for 100% (light green), 90% (green), and 68% (dark green)

**Fig. 4:**
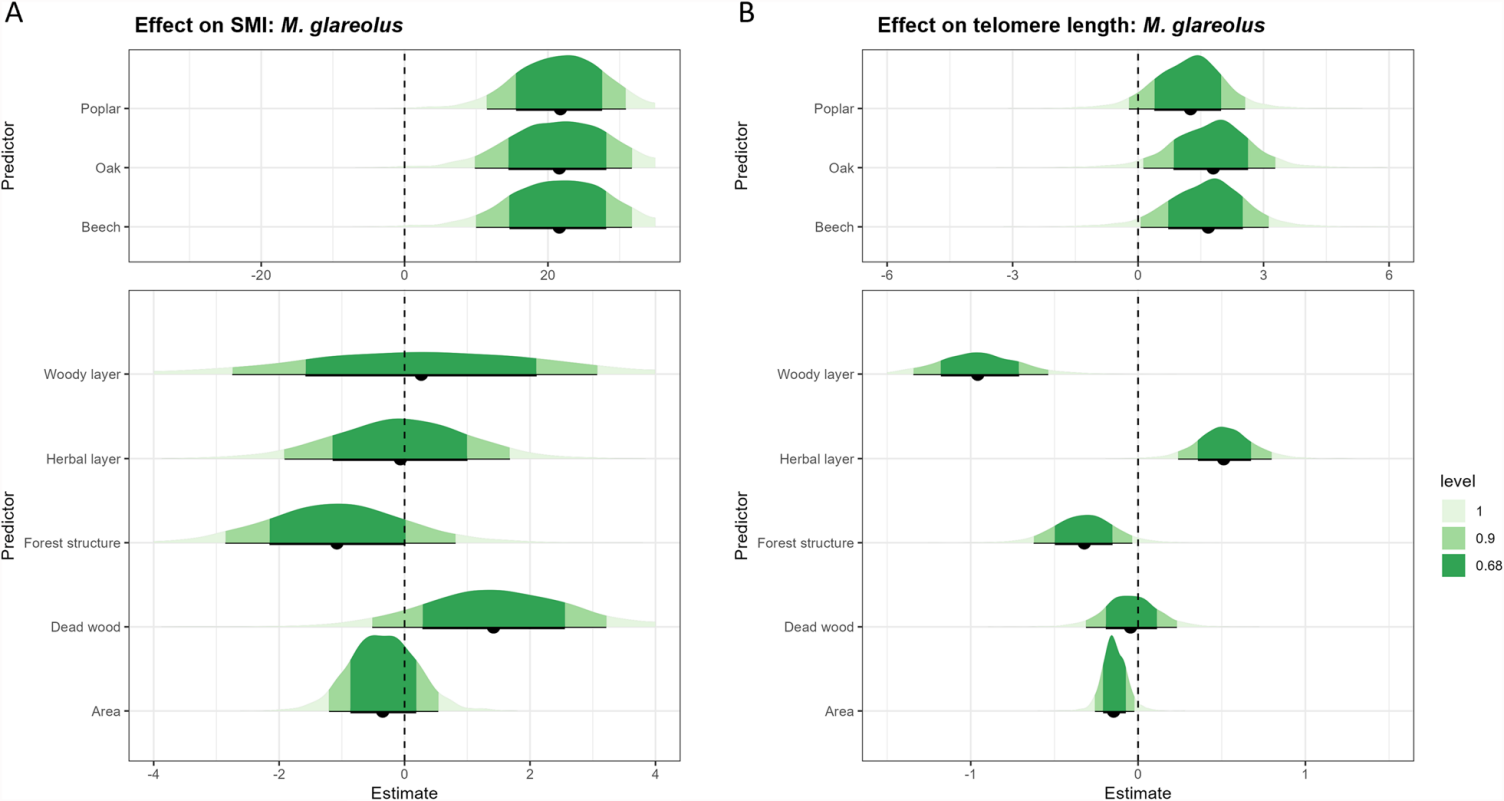
The effect of forest type (top panel); the four sub-indices of the structural complexity index and forest area (bottom panel) on individual body condition parameters in *Myodes glareolus*: **A** scaled mass index and **B** telomere length. Fig. 2 caption provides interpretation guidance. The credible interval is shown for 100% (light green), 90% (green), and 68% (dark green)

For *A. sylvaticus*, little evidence (68% CI) for a positive effect of the woody layer score on SMI was found (Fig. 3a). Our data showed little evidence (68% CI) for a negative effect of SCI on the telomere length of *A. sylvaticus* (Additional File 1: Supplementary Fig. S4b). When looking at the sub-indices, we found little evidence (68% CI) for a negative effect of both forest structure and woody layer, while herbal layer had a positive effect (Fig. 3b). For *M. glareolus*, we found little evidence (68% CI) for a positive effect of dead wood on SMI (Fig. 4a). For telomere length, our results showed very strong evidence (95% CI not containing zero) for a positive effect of herbal layer and a negative effect of woody layer. We also found weak evidence (90% CI) for a negative effect of forest structure (Fig. 4b). No statistically clear effect of SCI was found on SMI or telomere length (Additional File 1: Supplementary Fig. S5).

### Effect of individual body condition parameters on the likelihood of pathogen infection and tick presence

The likelihood of having at least 1 of the 11 pathogens present was higher in *A. sylvaticus* (19%) compared with *M. glareolus* (14%). For *Anaplasma phagocytophilum* more *M. glareolus* (4%) were infected than *A. sylvaticus* (< 1%); the low prevalence in *A. sylvaticus* was insufficient to analyze the effects of SMI and telomere length. In contrast, more *A. sylvaticus* were infected with *R. helvetica* (9%) compared with *M. glareolus* (5%). We also observed a higher prevalence of *B*. *burgdorferi* s.l. in *A. sylvaticus* (5%) compared with *M. glareolus* (2%), and *Spiroplasma ixodetis* was also more prevalent in *A. sylvaticus* (6%) than in *M. glareolus* (3%). In addition, a greater proportion of *A. sylvaticus* individuals had at least one tick (83%) compared with *M. glareolus* (47%), and *A. sylvaticus* had a higher average number of ticks per individual (4 ± 5) than *M*. *glareolus* (1 ± 2). Therefore, we used the number of ticks for *A. sylvaticus* and the presence/absence of ticks for *M. glareolus* in the paragraph below (effect of the other variable per species is shown in Additional File 1: Supplementary Fig. S6).

A total of 629 ticks were removed from the captured individuals. The majority were larvae (611), and some nymphs (8) and adult females (4) were removed. Some ticks (6) were too damaged to identify the life stage. Most ticks belonged to the species *Ixodes ricinus* (500), additionally, some *I. trianguliceps* (50) were found. Some ticks were found to belong to the genus *Ixodes* but were too damaged to identify the species (55), and other ticks were too damaged to identify even to the genus level (24).

**Fig. 5:**
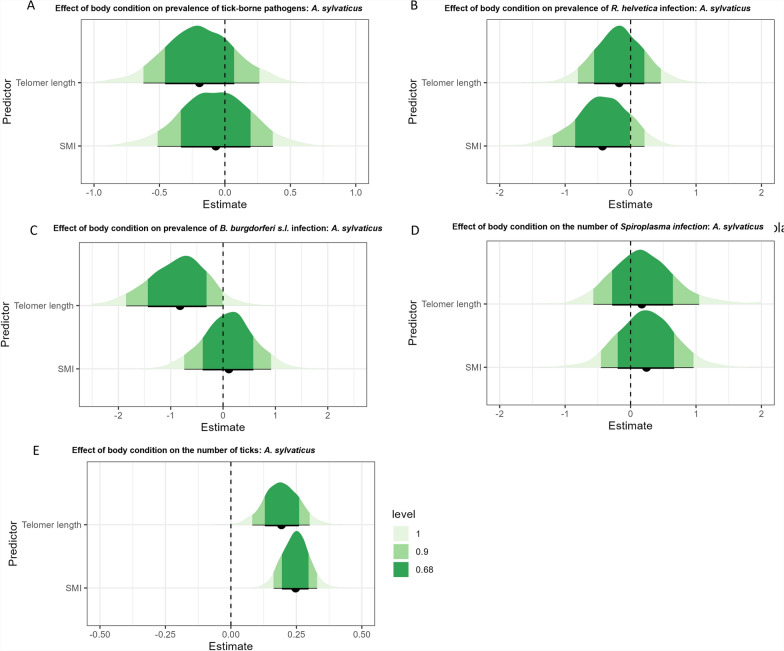
The effect of the two body condition parameters on the likelihood of a pathogen infection in *Apodemus sylvaticus*: **A** for any of the 11 pathogens, **B** *Rickettsia helvetica*, **C** *Borrelia burgdorferi* sensu lato, **D** *Spiroplasma ixodetis*, and **E** the number of ticks per individual. The graphs show the estimated statistical effect of each of the parameters. The credible interval is shown for 100% (light green), 90% (green), and 68% (dark green)

**Fig. 6:**
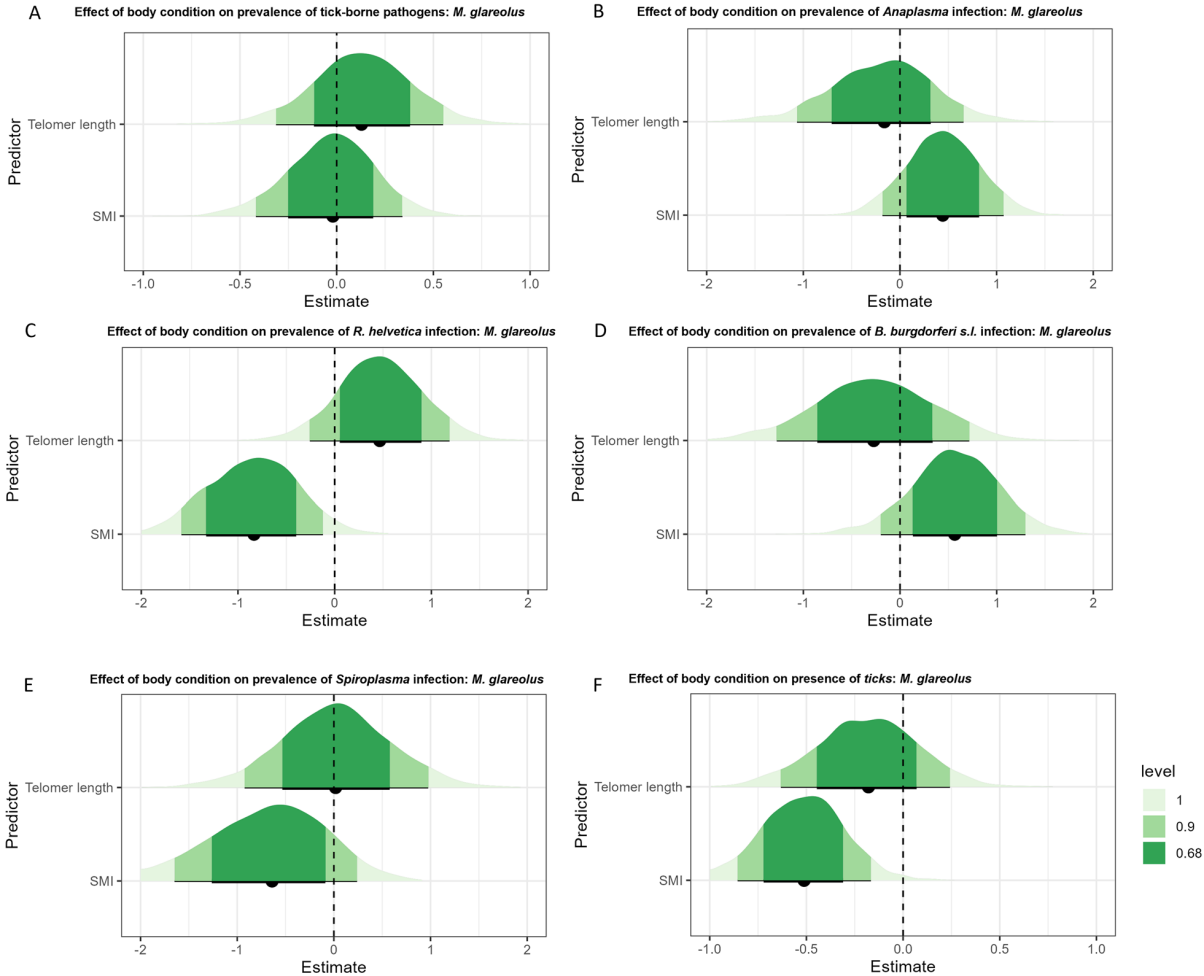
The effect of the two body condition parameters on the likelihood of a pathogen infection in *Myodes glareolus*: **A** for any of the 11 pathogens, **B** *Anaplasma phagocytophilum*, **C** *Rickettsia helvetica*, **D** *Borrelia burgdorferi* sensu lato, **E** *Spiroplasma ixodetes*, and **F** the presence of ticks on an individual. The graphs show the estimated effect of each of the parameters. The credible interval is shown for 100% (light green), 90% (green), and 68% (dark green)

In *A. sylvaticus*, our analysis revealed that neither telomere length nor SMI had a statistically significant effect on the likelihood of infection with at least 1 of the 11 pathogens examined (Fig. 5a). However, our data showed little evidence (68% CI) for a decrease in the prevalence of *R. helvetica* in *A. sylvaticus* with increasing SMI (Fig. 5b). The results also showed weak evidence (90% CI) for a negative relationship between the probability that an *A. sylvaticus* was infected with *B. burgdorferi *sensu stricto and telomere length (Fig. 5c). No clear effect of SMI or telomere length was found on the prevalence of *Spiroplasma* (Fig. 5d). Very strong evidence (95% CI not containing zero) was found for a positive effect of both SMI and telomere on the number of ticks per individual (Fig. 5e). In *M. glareolus*, we found no clear effect on the likelihood of infection with at least 1 of the 11 pathogens (Fig. 6a). Data showed little evidence (68% CI) for a positive effect of SMI on the prevalence of *Anaplasma* and *B. burgdorferi* s.l. (Fig. 6b, d). For *R. helvetica*, results showed little evidence (68%) for a positive effect of telomere length and weak evidence (90% CI) for a negative effect of SMI (Fig. 6c). Little evidence (68% CI) was found for a negative relationship between SMI and the chance of being infected with *Spiroplasma* (Fig. 6e). Lastly, weak evidence was found for a negative relationship between SMI and the likelihood of tick presence on *M. glareolus* (Fig. 6f).

## Discussion

In this study, we analyzed the relationship between forest structural complexity and both the density of small mammals and the individual body condition, and how body condition in turn affects the infection prevalence. We expected that the density of small mammals would increase with increasing structural complexity because more complex forests are believed to provide more food availability and niches for individuals to co-exist [6]. We also expected individuals to be in better body condition owing to the higher food availability and more cover from the understory vegetation against predation. Individuals with better body condition were also expected to have a lower prevalence of pathogens. This was assumed because these individuals have a higher energy reserve to allocate to their immune system.

### Higher forest structural complexity leads to an increase in density of small mammals

Rodent abundance is often used to evaluate the habitat quality, and it is assumed that the density increases with better quality [6, 12]. We used the SCI and its four sub-indices to assess which aspects of forest complexity influence the abundance of small mammals [49]. Our results showed a positive effect of SCI on total small mammal captures, aligning with our hypothesis and previous studies [9, 12, 64].

When looking more closely at the four sub-indices, we found that herbal layer and dead wood had a positive effect on the number of captured individuals, while woody layer, forest structure, and forest area had a negative effect. These effects were more pronounced in bank voles compared with the wood mice. Wood mice are a more generalist species in comparison with bank voles; it is thus possible that they also use the surrounding crop and grasslands [18]. The positive effect of the amount of dead wood and the cover of the understory vegetation is to be expected. They both provide cover against predation [18, 19] but can also be used as a food source such as leaves, seeds, fruits, and insects [10, 12]. Previous studies also found that the density of small mammals increased with increasing understory vegetation and the amount of dead wood mammals [9, 12, 64]. The negative effect of the forest structure and woody layer sub-indices was not expected. However, small mammals seem to prefer the conditions of younger forests, and forest scoring high on the woody layer and forest structure sub-index are overall older forests [18].

The relationship between forest area and density was negative, meaning that more individuals were captured in smaller forests. This is in line with previous research and is possibly an edge effect [65–67]. Smaller forests have a higher edge-to-interior ratio, and small mammals seem to do well in forest edges. Lastly, we found the highest number of captured individuals in oak, then beech, and lastly poplar forests. Acorns are preferred over beech nuts by small mammals [68]. It is possible that the food availability is highest in oak forests, allowing them to support higher densities of small mammals. Previous research supports this idea, demonstrating that small mammal densities are indeed higher in oak forests compared with beech and poplar forests [68, 69].

### Forest structural complexity has a positive effect on SMI

Body condition, influenced by food availability and predation pressure, was expected to improve with increased forest complexity, reflected in higher SMI and longer telomere lengths.

In this study, we found a positive relationship between SCI and SMI for both *A. sylvaticus* and *M. glareolus*, with *A. sylvaticus* benefiting from the woody layer and *M*. *glareolus* from dead wood. A preference of *M. glareolus* for dead wood has previously been found [70]. Dead wood provides both cover and food availability, as bank voles are known to eat fungi [71]. Similarly, for *A. sylvaticus*, the woody layer, characterized by tree and shrub species composition and diameter, likely increases food availability and cover, which in turn improves their body condition.

Telomere length results were more complex. Both species showed positive responses to the herbal layer, but negative effects from the woody layer and forest structure. *M. glareolus* also had shorter telomeres in larger forests, while *A. sylvaticus* exhibited a negative relationship with SCI. Contrary to our expectations, individuals from forests with higher SCI had shorter telomeres. As expected, the positive impact of the herbal layer on telomere length may be attributed to increased food availability and cover, which likely reduces chronic stress. Previous research also showed that individuals in a habitat with more cover and lower predation risk were less stressed [72, 73]. However, forests with high scores for structure and woody layer—characterized by 51–75% canopy cover, uneven-aged stands, and the presence of large trees (DBH > 80 cm)—may create canopy gaps, potentially increasing predation pressure and, consequently, stress levels. This could explain the observed negative effects on telomere length in these forests. Higher competition through increased density could also lead to more stress, leading to shorter telomeres [74]. Since telomeres shorten by both stress and age [24, 58], it is also possible that individuals with shorter telomeres are not more stressed but are older individuals. This would mean that the life expectancy of individuals is higher in more complex forests. Initially, we considered telomere length as an indicator of stress, assuming that age would have a minimal effect in these short-lived animals. However, based on our results, it appears more likely that telomere length primarily reflects age rather than stress in these individuals. However, we were not able to determine the age of the individuals on the basis of morphology, we could only distinguish juveniles and adults.

### The effect of body condition on the likelihood of pathogen infection depends on the pathogen and host species

Individuals with a better body condition are expected to be less susceptible to pathogens because they have more resources to allocate to their immune system. Therefore, we expected that individuals with higher SMI and longer telomeres would have a lower prevalence of pathogens. However, we did not find a relationship between either SMI and telomere length and the chance of being infected with at least 1 of the 11 pathogens tested. While the prevalence of certain pathogens did demonstrate a relationship with SMI and/or telomere length, the effects varied depending on the specific pathogen and host species. Consequently, combining all pathogens may obscure any clear relationships.

The prevalence of *Borrelia burgdorferi* s.l., the complex of bacteria that causes Lyme borreliosis in humans, increased with increasing SMI and with decreasing telomere length in *M. glareolus*. This means that heavier and more stressed (or older) individuals were more often infected with *Borrelia burgdorferi* s.l.. Since we also found that individuals in more structural complex forests have higher SMI and shorter telomere lengths, this would mean that the prevalence of *Borrelia burgdorferi* s.l. is also higher in more structural complex forests. However, we did not find this increased prevalence with structural complexity [75]. This would be disadvantageous since forest management is focused on increasing forest structural complexity for the many benefits it brings. However, it must be noted that the prevalence of *B. burgdorferi* s.l. was relatively low, with only 2% of *M. glareolus* and 5% of *A. sylvaticus* infected. In a review by Homeester et al. [31], higher prevalence of *B. burgdorferi* s.l. was found, with 11% for *A. sylvaticus* and 21% for *M. glareolus*. However, Sluydts et al. [76] found similar pathogen prevalence for both species and that these were lower than other European countries (France, Germany, and Poland).

*Anaplasma phagocytophilum* and *Rickettsia helvetica* are also known to cause disease in humans. The prevalence of *R. helvetica* (7%) was higher than for *A. phagocytophilum* (3%). The prevalence of *R. helvetica* decreased with increasing SMI and decreasing telomere length, the opposite of *B. burgdorferi* s.l. While *A. phagocytophilum* did increase with increasing SMI. This suggests that some pathogens thrive when an individual’s body condition is better, while others may be more prevalent in individuals with poorer body condition. Such variability complicates efforts to correlate body condition with the likelihood of infection across all pathogens.

We also found that *A. sylvaticus* had a higher likelihood of being infested with ticks and, on average, harbored more ticks than *M. glareolus*. This may be attributed to *M. glareolus* developing resistance to ticks after repeated tick bites, whereas *A. sylvaticus* does not [77]. Individuals with lower SMI were more likely to have at least one tick. In *A. sylvaticus*, we identified a negative relationship between the presence of ticks and telomere length, suggesting that individuals in poorer body condition are more susceptible to tick bites. Most research, however, looks at the number of ticks, or tick burden. For *M. glareolus*, no effect of SMI or telomere length was found on the number of ticks. Conversely, in *A. sylvaticus*, individuals with longer telomeres and higher SMI exhibited greater tick numbers. This suggests that individuals in better condition may experience higher tick encounters. Heavier individuals generally have larger home ranges and, thus, higher encounter rates with ticks [78–80].

### Limitations and recommendations for further research

Small mammals were trapped for a single night per plot; therefore, it is possible that weather conditions or local disturbances influenced our capture success. Moreover, the captures took place in one season. It is known that densities of rodents and pathogen prevalence can fluctuate over seasons and years [81–83]. Sampling small mammals during different seasons and multiple years would make it possible to also study temporal variations.

We used telomere length as a measure of stress, with shorter telomeres indicating higher chronic stress. However, telomere length is both influenced by stress and ageing. Our results rather point in the direction of an age effect instead of stress effect on telomere length since individuals in more complex forests had shorter telomeres. It seems more plausible that these longer telomere lengths are the effect of old age rather than high chronic stress. Therefore, we recommend using a different parameter for stress than telomere length.

Most studies on the condition of small mammals focus on population level, looking at the individual-level, as done in this research, could give more insight on how structural complexity influences the condition of small mammals. Many studies on small mammals compare two conditions, such as the comparison between the presence and absence of dead wood, conifers and broadleaved tree species, forests with and without disturbance, among others. [29, 84–91]. The structural complexity index makes it possible to compare changes over a composite gradient. The four sub-indices also provide the possibility to look in more detail at which aspects of the structural complexity have the largest impact.

## Conclusions

Our study highlights that increasing forest structural complexity improves small mammal population densities and individual body condition, primarily owing to enhanced food and shelter provided by the herb layer and dead wood. Forest management practices focused on increasing multifunctionality and biodiversity in forest through increased structural complexity also has a positive effect on the density and the physical condition of small mammals. However, improved body condition in more complex forests is linked to a higher prevalence of *Borrelia burgdorferi* (Lyme disease pathogen), despite the relatively low prevalence (3%). This higher risk stems from better small mammal condition and increased population densities in complex forests. While structural complexity supports biodiversity and mammal condition, management plans should address the associated disease risks, such as reducing human–tick contact through wider paths and avoiding overhanging vegetation.

## Supplementary Information

Additional File 1

## Data Availability

The data supporting the findings of the study are available within the article and its supplementary materials.

## Abbreviations

SMI: Scaled mass index
SCI: Structural Complexity Index
CI: Credible interval
DBH: Diameter at breast height
