# Supplementary material for: Influence of forest structural complexity on small mammal body condition and its impact on tick burden and pathogen prevalence
Source: Parasit Vectors. 2025 Jun 18;18:227. doi: 10.1186/s13071-025-06874-0 (PMC12177995; doi:10.1186/s13071-025-06874-0)
Supplement: Supplementary file 1 — Additional File 1 [file 13071_2025_6874_MOESM1_ESM.docx]

**Influence of Forest Structural Complexity on Small Mammal Body Condition and Its Impact on Tick Burden and Pathogen Prevalence**

Tosca Vanroy, Lander Baeten_,_ An Martel, Bram Catfolis, Manoj Fonville, Luc Lens, Frank Pasmans, Hein Sprong, Diederik Strubbe, Elin Verbrugghe, Kris Verheyen

**Supplemental information**

**S1: Detailed description of plot characterization and Structural Complexity Index calculation**

In each forest a 1ha core plot was predetermined during the plot exploration. Around the centre of the core plot, we worked with three concentric circles with a radius of 4.5m, 9m and 18m (C_1_, C_2_ and C_3_ in Fig. S1). Two sampling lines were placed through the middle of the plot and perpendicular to each other (dashed lines in Fig. S1). Markers were placed at 7 and 18 meters from the centre (black dots in Fig. S1). The vegetation analysis was conducted in a 10 by 10m plot (square with dotted lines in Fig. S1). This was marked by the markers at 7m, so the 4 lines are the diagonals of the square. The sampling happened in different layers: tree layer, shrub layer and herb layer (height of less than 2m). For each species in each layer the percentage of cover was estimated. The total cover per layer was also estimated.

Each tree was assigned to a circle based on the distance between the tree and the centre. Then, the circumference of the tree was measured. If the circumference did not correspond with the desired circumference of that circle (C_1_ circumference > 22cm, C_2_: 22cm ≤ circumference < 122cm and C_3_: circumference ≥ 122 cm), the tree was not considered. The distance to the centre was measured using a Vertex and the circumference was measured twice at breast height, both measurements perpendicular to each other. Dead wood was measured using the Line Intersect Method (Marshall et al., 2003) using the 4 line transects of 18m each. For all lying dead trees and dead wood elements, the diameter at the intersection with the line transect was measured. Only the length of pieces with a diameter of at least 7cm were measured. For each piece of dead wood, the class of decay was noted.

Using the variables quantified during plot characterization, a forest Structural Complexity Index (SCI) was created. We used four predefined ‘sub-indices’ as clusters, i.e. grouping together variables related to either forest structure, the woody or herb layer, or to dead wood (Table 1). Next, to decide which variables per plot contributed most to the scores of each sub-index, we adopted the ‘threshold method’ (Manning et al., 2018), which counts the number of variables that exceeded a certain threshold. These threshold values were determined based on the distribution of the measured values from our 19 plots. For numerical variables, we calculated the median of the values (Table S1), and considered the ecological significance of these medians before using them as the threshold. For the ordinal values, we made a choice based on the ecological significance. If the measured value was smaller than or equal to the chosen ecological significance threshold, the variable was assigned a score of 0, when the score was larger than the threshold a score of 1 was used. Each sub-index, as well as each retained variable within a sub-index, was assigned equal weight in the total score (e.g. a weight of 0.2 if the sub-index contains five variables). So, each forest received a forest Structural Complexity Index (SCI) score varying between 0 and 1. A low score (closer to zero) indicates forests with a simpler structure while a high score (closer to 1) is characteristic of forests with a more complex structure.

**Table S1** Threshold for the different variables. The threshold values were determined based on the distribution of the values from our 19 plots. For the threshold of the numerical variables (N) we calculated the median of the values. We checked the ecological significance of the medians before using them as the threshold. For the ordinal values (O) we made a choice based on the ecological significance. If the value is smaller than the threshold it gets a score of 0 and when the score is equal to or larger than the threshold it gets a score of 1.

| **Sub-Index** | **Variables** | **Units** | **Threshold** |
| --- | --- | --- | --- |
| **Forest structure** | Canopy cover (O) | The percentage of canopy cover (canopy cover of 76-100% = 1; 51-75% = 2) | 1 |
|  | Forest age (O) | The stand age (1-60 years = 1, 61-100 years = 2, uneven aged = 3) | 2 |
|  | Tree layers (O) | The number of storeys | 1 |
|  | Horizontal structure (O) | The spatial tree species mixture (homogeneous = 1, individual = 2) | 1 |
| **Woody layer** | Tree species (N) | The number of tree species inside the plot | 2 |
|  | Large trees (N) | The number of trees with a diameter at breast height larger than 40cm | 11 |
|  | Very large trees (N) | The number of trees with a diameter at breast height larger than 80cm | 0 |
|  | Natural regeneration (N) | The number of tree species in the herb layer that are species in natural regeneration | 2 |
|  | SD DBH (N) | The standard deviation of the diameter at breast height | 16 |
| **Herbal layer** | Plant species (N) | The number of plant species in the herb layer | 10 |
|  | Degree of rareness (N) | The degree of rareness is based on the occurrence of a plant species in a 4 x4km grid | 6 |
|  | Total cover (N) | The percentage cover of the herb layer | 75 |
| **Dead wood** | Basal area standing DW (N) | The basal area of all standing dead trees (m²/ha) | 0.4 |
|  | SD diameter DW (N) | The standard deviation of the diameter at breast height of the standing dead trees | 0 |
|  | Large dead trees (N) | The number of standing dead trees with a diameter at breast height larger than 40cm | 0 |
|  | Length lying DW (N) | The sum of all lengths of the lying dead trees with a diameter larger than 40cm | 0 |
|  | Dead wood classes (O) | the number of dead wood diameter classes that occurred (class 1: 2-7cm, class 2: 7-22cm, class 3: 22-40cm, class 4: > 40cm) | 2 |

**Table S2** coordinates, area (m²), stand age (years) Structural complexity index (SCI) and the number of captured individuals of all selected forests.

| **Plot ID** | **Forest name** | **Coordinates** | **Area (m²)** | **Stand age (years)** | **SCI** | **# Captured individuals** | **# Ticks** |
| --- | --- | --- | --- | --- | --- | --- | --- |
| Beech 2 | Trimpontbos (Everbeekse bossen) | 50°45'43.4"N 3°46'33.1"O | 38271 | 61-100 | 0.18 | 13 | 43 |
| Beech 3 | Neigembos | 50°48'34.7"N 4°03'50.9"O | 95086 | Uneven | 0.20 | 10 | 12 |
| Beech 4 | Parkbos-Uilenbroek | 50°50'15.9"N 3°51'03.2"O | 131128 | 61-100 | 0.31 | 15 | 40 |
| Beech 5 | / | 50°48'16.4"N 3°43'26.4"O | 28843 | Uneven | 0.56 | 17 | 41 |
| Beech 6 | Hasseltbos | 50°49'34.3"N 3°52'26.2"O | 106662 | 61-100 | 0.30 | 7 | 24 |
| Beech 8 | Bos Ter Eecken | 50°46'29.8"N 3°39'21.9"O | 29995 | 61-100 | 0.36 | 11 | 101 |
| Beech 9 | Berchembos | 50°49'08.8"N 4°04'36.1"O | 81139 | 61-100 | 0.52 | 28 | 16 |
| Oak 3 | Spiegeldriesbos / Munkbos | 50°55'07.2"N 3°45'44.9"O | 109764 | Uneven | 0.39 | 21 | 36 |
| Oak 4 | Kluysbos | 50°45'38.1"N 3°56'31.9"O | 66855 | 61-100 | 0.40 | 23 | 61 |
| Oak 5 | Cotthembos | 50°54'38.3"N 3°51'17.4"O | 46335 | 61-100 | 0.25 | 32 | 68 |
| Oak 6 | Kartelobos | 50°55'50.9"N 4°11'54.3"O | 80513 | 61-100 | 0.22 | 11 | 9 |
| Oak 7 | Domein Gaasbeek | 50°47'37.4"N 4°12'00.3"O | 251472 | Uneven | 0.47 | 32 | 10 |
| Poplar 1 | / | 50°47'22.3"N 3°48'49.7"O | 43611 | 1-60 | 0.44 | 9 | 53 |
| Poplar 2 | Steenbergbos (Everbeekse bossen) | 50°46'46.3"N 3°46'39.7"O | 47482 | 1-60 | 0.69 | 3 | 4 |
| Poplar 3 | / | 50°55'38.9"N 3°54'01.9"O | 73873 | 1-60 | 0.64 | 13 | 28 |
| Poplar 5 | / | 50°45'05.2"N 3°49'40.4"O | 26052 | 1-60 | 0.55 | 29 | 41 |
| Poplar 6 | / | 50°54'53.4"N 4°07'29.2"O | 29770 | 1-60 | 0.35 | 25 | 23 |
| Poplar 7 | Oombergse bossen | 50°54'26.8"N 3°49'52.5"O | 60206 | 1-60 | 0.26 | 0 | 0 |
| Poplar 8 | / | 50°46'20.9"N 4°00'58.5"O | 48582 | 1-60 | 0.48 | 15 | 32 |


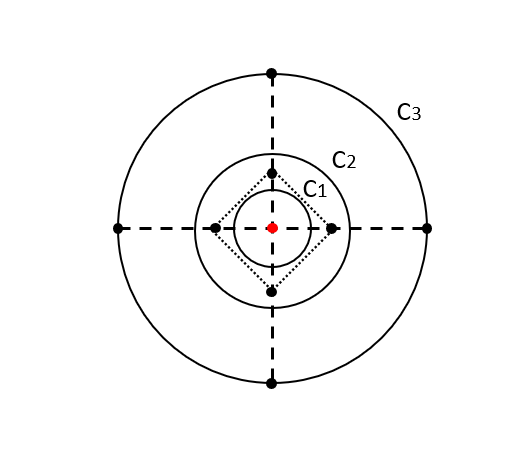


**Figure S1** Setup of the core plot. The core plot was made up by 3 concentric circles (C1, C2 and C3). Two sampling lines were placed perpendicular to each other through the centre of the plot (dashed lines). Four sticks were placed on these lines at 7 meters from the centre to form the 10 m by 10 m square in which the vegetation analysis was conducted (square in dotted lines). C1 is a circle with radius of 4.5 m, in which all trees with a circumference of less than 22 cm were measured. In C2, with a radius of 9 m, all trees with a circumference between 22 and 122 cm were measured. In C3, with a radius of 18 m, all trees with a circumference equal to or larger than 122 cm were measured (based on the protocol of the Flemish Forest Inventory (Govaere, 2019)).

**S2: Relative Telomere analysis**

DNA was extracted from ear tip biopsies of mice using the DNeasy Blood and Tissue Kit (Qiagen) according to the manufacturer’s protocol. The concentration and purity of the extracted DNA were assessed using a Nanodrop 1000 spectrophotometer.

Relative telomere length was determined using quantitative PCR (qPCR), following the method described by Cawthon (2002). The telomere length of each sample was calculated as the ratio (T/S) of telomere repeat copy number (T) to a single-copy control gene (S), relative to a reference sample. The reference sample consisted of a pooled DNA mix from all samples. Acidic ribosomal phosphoprotein PO (Rplp0) was used as the single-copy control gene (Callicott and Womack, 2006; Cawthon, 2002).

PCR reactions were performed in 25 µL volumes containing 5 µL of DNA (with varying concentrations for serial dilutions of the standard curve and a 1/75 dilution for unknown samples), 12.5 µL SsoAdvanced Universal SYBR Green Supermix (Bio-Rad), primers (TelF: 100 nM; TelR: 200 nM; Rplp0 F-R: 500 nM), and nuclease-free water. The thermal cycling profile for the telomere primers consisted of an initial denaturation step at 95°C for 10 min, followed by 40 cycles of 95°C for 15 s and 56°C for 30 s, with fluorescence signal acquisition at the end of the 56°C step. A melting curve analysis was performed by increasing the temperature from 65°C to 95°C in 0.5°C increments. For the Rplp0 primers, the thermal cycling profile consisted of an initial denaturation at 95°C for 10 min, followed by 40 cycles of 95°C for 30 s, 60°C for 30 s, and 72°C for 30 s, with signal acquisition at the end of the 72°C step. A melting curve was generated by increasing the temperature from 65°C to 95°C in 0.5°C increments.

Primer efficiency was evaluated by plotting a standard curve in each qPCR run and a 1/75 dilution of the pooled DNA was included as a reference sample. All samples, including those used for the standard curve, were analyzed in duplicate, and the average cycle threshold (Cq) values were used to calculate the T/S ratio. Samples with a Cq difference greater than 1.0 between technical replicates were excluded from further analysis. Prior to calculating the relative telomere length (RTL), interplate variation was corrected. RTL was calculated using the 2^(-ΔΔCt) method, where ΔΔCt = (Cq_telomere - Cq_Rplp0)_sample - (Cq_telomere - Cq_Rplp0)_reference sample (Cawthon et al., 2002). After calculation, RTL values were log-transformed to normalize the distribution of the data prior to statistical analysis.


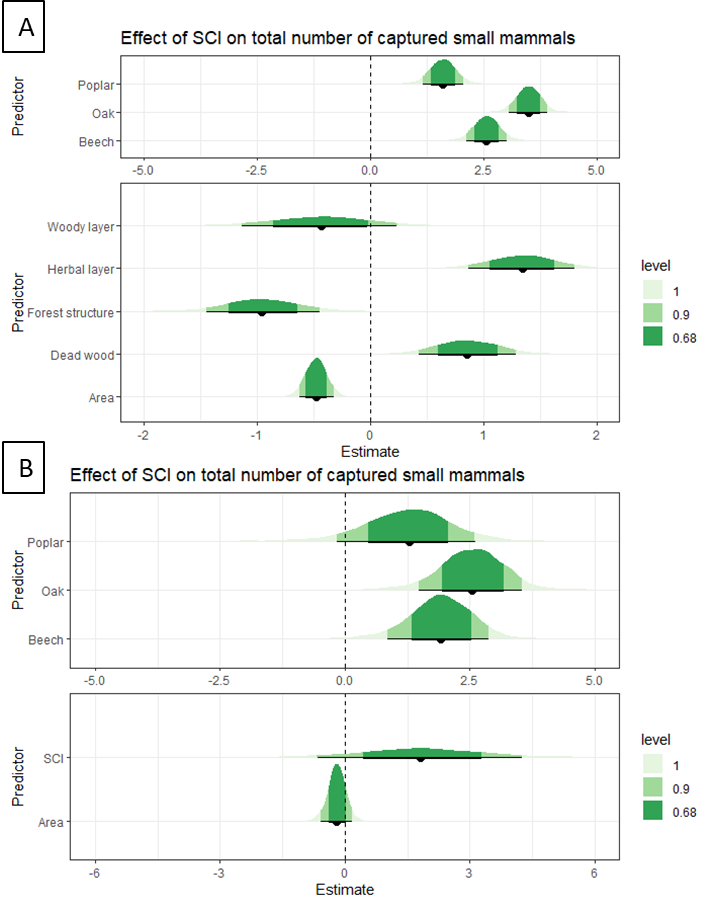


**Figure S2**: The effect of forest structural complexity (A), its four-subindices (B), and forest area on the number of captured individuals of both species combined. De upper graph is the estimated number of individuals captured for each dominant tree species for a forest of average size and all other parameters with a score of zero. The lower graph is the estimated effect of each of the parameters. The credible interval is shown for 100% (light green), 90% (green) and 68% (dark green).


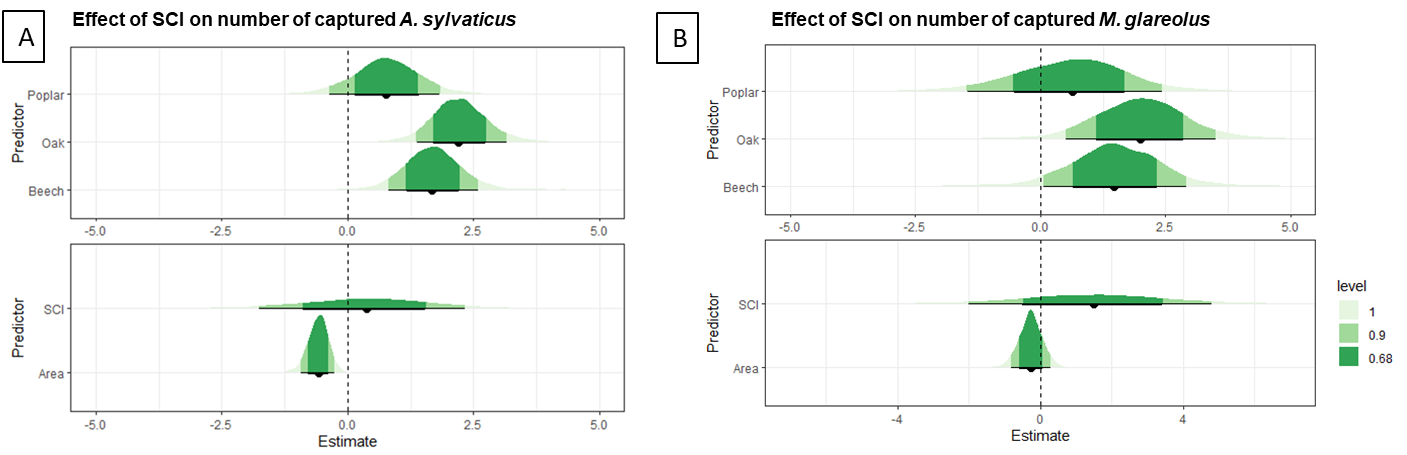


**Figure S3**: The effect of the structural complexity index and forest area on the number of captured individuals of each species: A) Apodemus sylvaticus and B) Myodes glareolus. De upper graph is the estimated number of individuals captured for each dominant tree species for a forest of average size and all other parameters with a score of zero. The lower graph is the estimated effect of each of the parameters. The credible interval is shown for 100% (light green), 90% (green) and 68% (dark green).


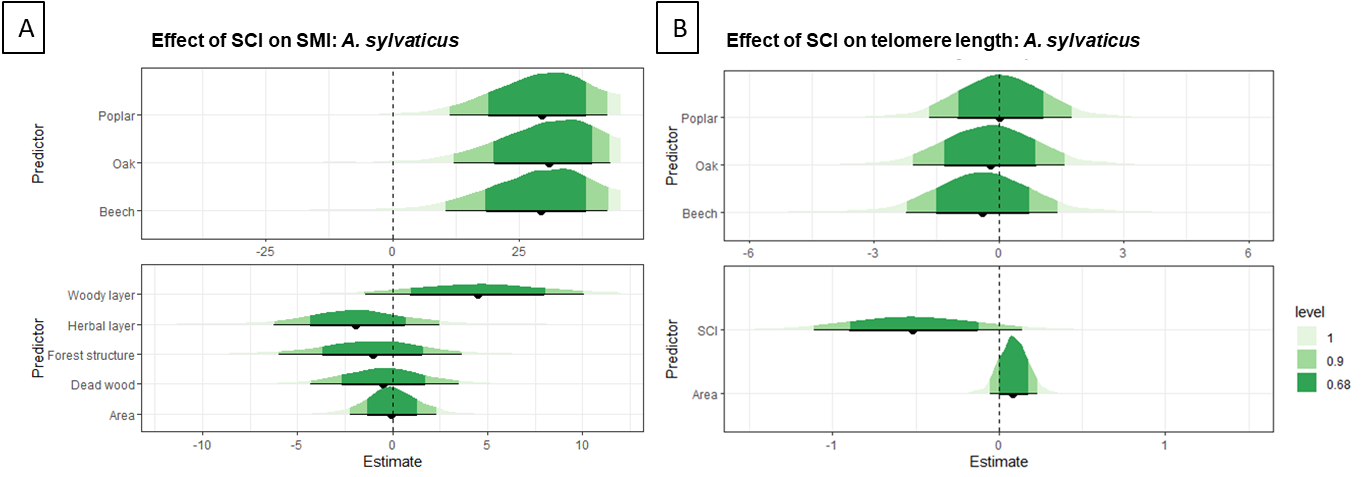


**Figure S4**: The effect of the structural complexity index and forest area on individual health parameters in Apodemus sylvaticus: (A) Scaled Mass Index and (B) telomere length. Top panel: the estimated value for a forest of average size and all other parameters with a score of zero. Bottom panel: the estimated effect of each of the parameters. The credible interval is shown for 100% (light green), 90% (green) and 68% (dark green).


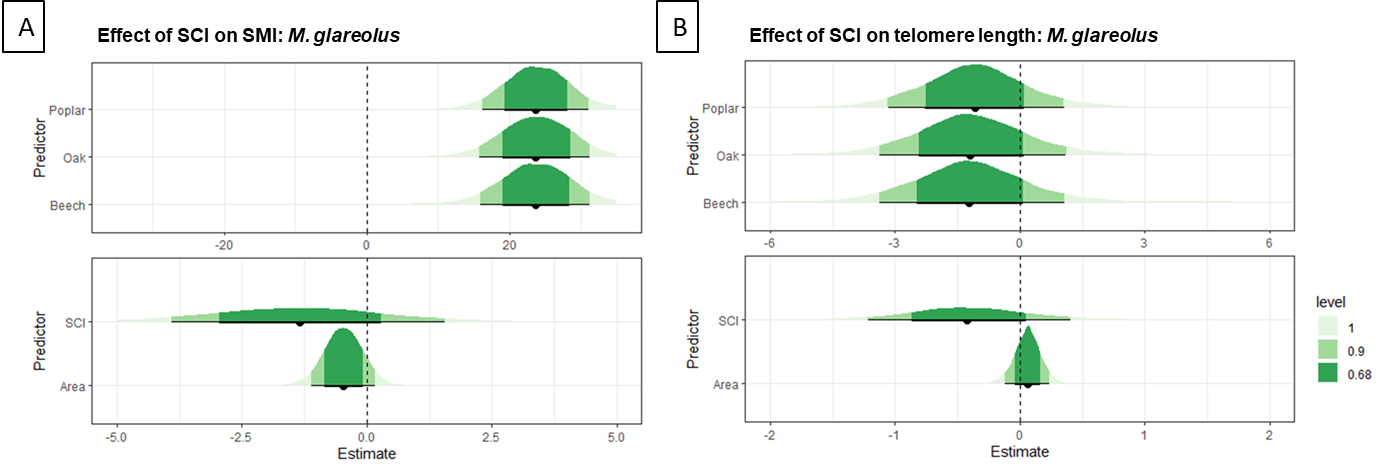


**Figure S5:** The effect of the structural complexity index and forest area on individual health parameters in Myodes glareolus: (A) Scaled Mass Index and (B) telomere length. Top panel: the estimated value for a forest of average size and all other parameters with a score of zero. Bottom panel: the estimated effect of each of the parameters. The credible interval is shown for 100% (light green), 90% (green) and 68% (dark green).


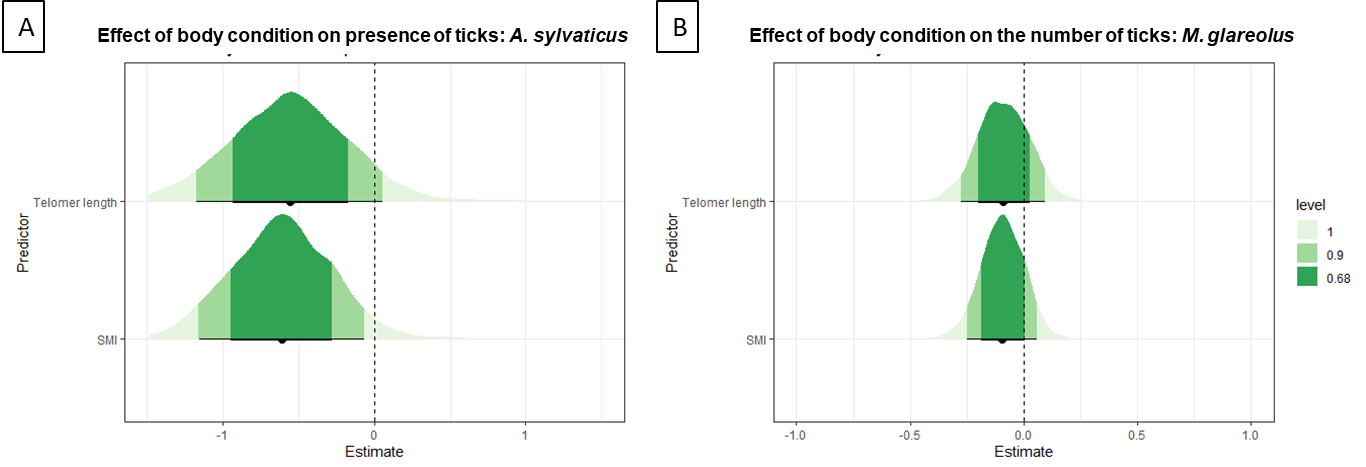


**Figure S6:** The effect of the two body condition parameters on (A) the presence of at least on tick on A. sylvaticus (B) the number of ticks on M. glareolus. The graphs show the estimated effect of each of the parameters. The credible interval is shown for 100% (light green), 90% (green) and 68% (dark green).

**Table S3** Parameter estimates and credible interval for the parameters of all models

| mouse_density ~ -1 + Area_log_norm + Forest_type + dead_wood + forest_structure  + herbal_layer + woody_layer | | | | | | | |
| --- | --- | --- | --- | --- | --- | --- | --- |
|  | **Estimate** | **Est.Error** | | **l-95% CI** | | **u-95% CI** | |
| Area_log_norm | -0.47 | | 0.09 | | -0.66 | | -0.29 |
| Forest_typeBeech | 2.58 | | 0.28 | | 2.03 | | 3.12 |
| Forest_typeOak | 3.50 | | 0.26 | | 2.99 | | 4.00 |
| Forest_typePoplar | 1.61 | | 0.27 | | 1.07 | | 2.14 |
| Dead_wood | 0.63 | | 0.26 | | 0.35 | | 1.37 |
| Forest_structure | -0.95 | | 0.30 | | -1.55 | | -0.36 |
| Herbal_layer | 1.35 | | 0.29 | | 0.78 | | 1.92 |
| Woody_layer | -0.44 | | 0.42 | | -1.27 | | 0.36 |
|  |  | |  | |  | |  |
| mouse_density ~ -1 + Area_log_norm + Forest_type + SCI + (1 \| plotID) | | | | | | | |
|  | **Estimate** | | **Est.Error** | | **l-95% CI** | | **u-95% CI** |
| Area_log_norm | -0.19 | | 0.22 | | -0.66 | | 0.25 |
| Forest_typeBeech | 1.92 | | 0.62 | | 0.61 | | 3.06 |
| Forest_typeOak | 2.55 | | 0.64 | | 1.19 | | 3.77 |
| Forest_typePoplar | 1.26 | | 0.85 | | -0.52 | | 2.87 |
| SCI | 1.87 | | 1.54 | | -1.11 | | 5.08 |
|  |  | |  | |  | |  |
| density_woodmice ~ -1 + Area_log_norm + Forest_type + dead_wood + forest_structure + herbal_layer + woody_layer | | | | | | | |
|  | **Estimate** | | **Est.Error** | | **l-95% CI** | | **u-95% CI** |
| Area_log_norm | -0.50 | | 0.15 | | -0.81 | | -0.21 |
| Forest_typeBeech | 1.65 | | 0.47 | | 0.75 | | 2.59 |
| Forest_typeOak | 2.15 | | 0.43 | | 1.33 | | 2.97 |
| Forest_typePoplar | 0.89 | | 0.42 | | 0.04 | | 1.70 |
| Dead_wood | 1.06 | | 0.41 | | 0.25 | | 1.89 |
| Forest_structure | -0.13 | | 0.49 | | -1.05 | | 0.86 |
| Herbal_layer | 0.06 | | 0.51 | | -0.95 | | 1.03 |
| Woody_layer | -0.20 | | 0.69 | | -1.57 | | 1.14 |
|  |  | |  | |  | |  |
| density_woodmice ~ -1 + Area_log_norm + Forest_type + SCI + (1 \| plotID) | | | | | | | |
|  | **Estimate** | | **Est.Error** | | **l-95% CI** | | **u-95% CI** |
| Area_log_norm | -0.58 | | 0.21 | | -1.02 | | -0.19 |
| Forest_typeBeech | 1.70 | | 0.55 | | 0.66 | | 2.80 |
| Forest_typeOak | 2.23 | | 0.56 | | 1.18 | | 3.38 |
| Forest_typePoplar | 0.77 | | 0.68 | | -0.61 | | 2.11 |
| SCI | 0.34 | | 1.31 | | -2.36 | | 2.74 |
|  |  | |  | |  | |  |
| density_bankvole ~ -1 + Area_log_norm + Forest_type + dead_wood + forest_structure + herbal_layer + woody_layer | | | | | | | |
|  | **Estimate** | | **Est.Error** | | **l-95% CI** | | **u-95% CI** |
| Area_log_norm | -0.44 | | 0.12 | | -0.69 | | -0.20 |
| Forest_typeBeech | 1.94 | | 0.36 | | 1.22 | | 2.64 |
| Forest_typeOak | 3.22 | | 0.35 | | 2.53 | | 3.91 |
| Forest_typePoplar | 0.97 | | 0.36 | | 0.26 | | 1.65 |
| Dead_wood | 0.68 | | 0.35 | | -0.00 | | 1.38 |
| Forest_structure | -1.50 | | 0.39 | | -2.25 | | -0.73 |
| Herbal_layer | 2.11 | | 0.36 | | 1.40 | | 2.83 |
| Woody_layer | -0.50 | | 0.56 | | -1.60 | | 0.59 |
|  |  | |  | |  | |  |
| density_bankvole ~ -1 + Area_log_norm + Forest_type + SCI + (1 \| plotID) | | | | | | | |
|  | **Estimate** | | **Est.Error** | | **l-95% CI** | | **u-95% CI** |
| Area_log_norm | -0.26 | | 0.34 | | -0.95 | | 0.41 |
| Forest_typeBeech | 1.49 | | 0.90 | | -0.30 | | 3.24 |
| Forest_typeOak | 2.00 | | 0.93 | | 0.14 | | 3.86 |
| Forest_typePoplar | 0.57 | | 1.21 | | -1.98 | | 2.85 |
| SCI | 1.54 | | 2.21 | | -2.96 | | 5.99 |
|  |  | |  | |  | |  |
| Bank voles: SMI ~ -1 + Area_log_norm + Forest_type + SCI + (1 \| plotID) | | | | | | | |
|  | **Estimate** | | **Est.Error** | | **l-95% CI** | | **u-95% CI** |
| Area_log_norm | -0.46 | | 0.40 | | -1.27 | | 0.31 |
| Forest_typeBeech | 23.87 | | 5.04 | | 14.32 | | 34.15 |
| Forest_typeOak | 23.91 | | 5.08 | | 14.17 | | 34.08 |
| Forest_typePoplar | 23.89 | | 4.75 | | 14.65 | | 33.49 |
| SCI | -1.38 | | 1.78 | | -4.87 | | 2.17 |
|  |  | |  | |  | |  |
| Bank voles: SMI ~ -1 + Area_log_norm + Forest_type + dead_wood + forest_structure + herbal_layer + woody_layer + (1 \| plotID) | | | | | | | |
|  | **Estimate** | | **Est.Error** | | **l-95% CI** | | **u-95% CI** |
| Area_log_norm | -0.34 | | 0.54 | | -1.41 | | 0.73 |
| Forest_typeBeech | 21.82 | | 7.25 | | 7.36 | | 35.84 |
| Forest_typeOak | 21.84 | | 7.37 | | 7.19 | | 36.34 |
| Forest_typePoplar | 21.84 | | 6.37 | | 9.24 | | 34.28 |
| Dead_wood | 1.45 | | 1.22 | | -0.93 | | 3.90 |
| Forest_structure | -1.09 | | 1.14 | | -3.28 | | 1.20 |
| Herbal_layer | -0.07 | | 1.11 | | -2.33 | | 2.08 |
| Woody_layer | 0.31 | | 2.03 | | -3.66 | | 4.20 |
|  |  | |  | |  | |  |
| Bank voles: Telomerlength ~ -1 + Area_log_norm + Forest_type + SCI + (1 \| plotID) | | | | | | | |
|  | **Estimate** | | **Est.Error** | | **l-95% CI** | | **u-95% CI** |
| Area_log_norm | 0.06 | | 0.11 | | -0.16 | | 0.28 |
| Forest_typeBeech | -1.19 | | 1.37 | | -3.79 | | 1.61 |
| Forest_typeOak | -1.16 | | 1.37 | | -3.79 | | 1.67 |
| Forest_typePoplar | -1.07 | | 1.30 | | -3.63 | | 1.68 |
| SCI | -0.41 | | 0.49 | | -1.37 | | 0.60 |
|  |  | |  | |  | |  |
| Bank voles: Telomerlength ~ -1 + Area_log_norm + Forest_type + dead_wood + forest_structure + herbal_layer + woody_layer + (1 \| plotID) | | | | | | | |
|  | **Estimate** | | **Est.Error** | | **l-95% CI** | | **u-95% CI** |
| Area_log_norm | -0.14 | | 0.08 | | -0.28 | | 0.01 |
| Forest_typeBeech | 1.64 | | 0.99 | | -0.44 | | 3.54 |
| Forest_typeOak | 1.75 | | 1.00 | | -0.31 | | 3.65 |
| Forest_typePoplar | 1.21 | | 0.88 | | -0.59 | | 2.96 |
| Dead_wood | -0.04 | | 0.17 | | -0.38 | | 0.29 |
| Forest_structure | -0.33 | | 0.18 | | -0.69 | | 0.03 |
| Herbal_layer | 0.52 | | 0.17 | | 0.17 | | 0.87 |
| Woody_layer | -0.96 | | 0.26 | | -1.47 | | -1.47 |
|  |  | |  | |  | |  |
| Wood mice: SMI ~ -1 + Area_log_norm + Forest_type + SCI + (1 \| plotID) | | | | | | | |
|  | **Estimate** | | **Est.Error** | | **l-95% CI** | | **u-95% CI** |
| Area_log_norm | -1.11 | | 1.01 | | -3.16 | | 0.84 |
| Forest_typeBeech | 31.74 | | 12.85 | | 7.12 | | 57.44 |
| Forest_typeOak | 33.69 | | 12.93 | | 8.77 | | 59.75 |
| Forest_typePoplar | 31.29 | | 12.07 | | 7.89 | | 55.37 |
| SCI | -3.00 | | 4.36 | | -11.40 | | 5.65 |
|  |  | |  | |  | |  |
| Wood mice: SMI ~ -1 + Area_log_norm + Forest_type + dead_wood + forest_structure + herbal_layer + woody_layer + (1 \| plotID) | | | | | | | |
|  | **Estimate** | | **Est.Error** | | **l-95% CI** | | **u-95% CI** |
| Area_log_norm | -0.01 | | 1.39 | | -2.79 | | 2.73 |
| Forest_typeBeech | 15.41 | | 18.58 | | -21.48 | | 52.21 |
| Forest_typeOak | 17.89 | | 18.71 | | -19.65 | | 55.05 |
| Forest_typePoplar | 17.59 | | 16.74 | | -16.42 | | 51.01 |
| Dead_wood | -0.46 | | 2.37 | | -5.26 | | 4.30 |
| Forest_structure | -1.06 | | 2.99 | | -7.24 | | 5.00 |
| Herbal_layer | -1.91 | | 2.82 | | -7.55 | | 3.66 |
| Woody_layer | 4.87 | | 4.10 | | -3.05 | | 13.57 |
|  |  | |  | |  | |  |
| Wood mice: Telomerlength ~ -1 + Area_log_norm + Forest_type + SCI + (1 \| plotID) | | | | | | | |
|  | **Estimate** | | **Est.Error** | | **l-95% CI** | | **u-95% CI** |
| Area_log_norm | 0.09 | | 0.09 | | -0.09 | | 0.27 |
| Forest_typeBeech | -0.39 | | 1.15 | | -2.70 | | 1.92 |
| Forest_typeOak | -0.22 | | 1.15 | | -2.48 | | 2.05 |
| Forest_typePoplar | 0.04 | | 1.08 | | -2.12 | | 2.13 |
| SCI | -0.52 | | 0.40 | | -1.30 | | 0.27 |
|  |  | |  | |  | |  |
| Wood mice: Telomerlength ~ -1 + Area_log_norm + Forest_type + dead_wood + forest_structure + herbal_layer + woody_layer + (1 \| plotID) | | | | | | | |
|  | **Estimate** | | **Est.Error** | | **l-95% CI** | | **u-95% CI** |
| Area_log_norm | -0.04 | | 0.11 | | -0.26 | | 0.18 |
| Forest_typeBeech | 1.41 | | 1.53 | | -1.61 | | 4.35 |
| Forest_typeOak | 1.66 | | 1.54 | | -1.37 | | 4.68 |
| Forest_typePoplar | 1.56 | | 1.37 | | -1.17 | | 4.22 |
| Dead_wood | -0.17 | | 0.19 | | -0.54 | | 020 |
| Forest_structure | -0.39 | | 0.25 | | -0.88 | | 0.17 |
| Herbal_layer | 0.24 | | 0.22 | | -0.21 | | 0.69 |
| Woody_layer | -0.52 | | 0.32 | | -1.15 | | 0.10 |
|  |  | |  | |  | |  |
| Bank voles: pathogen_presence ~ SMI_norm + TelomerLength_norm + (1 \| plotID) | | | | | | | |
|  | **Estimate** | | **Est.Error** | | **l-95% CI** | | **u-95% CI** |
| Intercept | -1.90 | | 0.28 | | -2.49 | | -1.39 |
| SMI_norm | -0.03 | | 0.23 | | -0.50 | | 0.40 |
| TelomerLength_norm | 0.13 | | 0.26 | | -0.40 | | 0.63 |
|  |  | |  | |  | |  |
| Bank voles: Anaplasma ~ SMI_norm + TelomerLength_norm + (1 \| plotID) | | | | | | | |
|  | **Estimate** | | **Est.Error** | | **l-95% CI** | | **u-95% CI** |
| Intercept | -4.40 | | 1.03 | | -6.95 | | -2.90 |
| SMI_norm | 0.45 | | 0.38 | | -0.29 | | 1.18 |
| TelomerLength_norm | -0.19 | | 0.54 | | -1.33 | | 0.84 |
|  |  | |  | |  | |  |
| Bank voles: Spiroplasma ~ SMI_norm + TelomerLength_norm + (1 \| plotID) | | | | | | | |
|  | **Estimate** | | **Est.Error** | | **l-95% CI** | | **u-95% CI** |
| Intercept | -4.63 | | 1.24 | | -8.15 | | -3.04 |
| SMI_norm | -0.71 | | 0.63 | | -2.07 | | 0.39 |
| TelomerLength_norm | 0.02 | | 0.58 | | -1.14 | | -1.14 |
|  |  | |  | |  | |  |
| Bank voles: R.helvetica ~ SMI_norm + TelomerLength_norm + (1 \| plotID) | | | | | | | |
|  | **Estimate** | | **Est.Error** | | **l-95% CI** | | **u-95% CI** |
| Intercept | -3.69 | | 0.73 | | -5.41 | | -2.53 |
| SMI_norm | -0.88 | | 0.49 | | -1.91 | | 0.02 |
| TelomerLength_norm | 0.47 | | 0.43 | | -0.37 | | 1.33 |
|  |  | |  | |  | |  |
| Bank voles: B. burgdorferi s.l. ~ SMI_norm + TelomerLength_norm + (1 \| plotID) | | | | | | | |
|  | **Estimate** | | **Est.Error** | | **l-95% CI** | | **u-95% CI** |
| Intercept | -4.85 | | 0.93 | | -6.99 | | -3.41 |
| SMI_norm | 0.56 | | 0.46 | | -0.39 | | 1.44 |
| TelomerLength_norm | -0.27 | | 0.61 | | -1.52 | | 0.86 |
|  |  | |  | |  | |  |
| Bank voles: tick presence ~ SMI_norm + TelomerLength_norm + (1 \| plotID) | | | | | | | |
|  | **Estimate** | | **Est.Error** | | **l-95% CI** | | **u-95% CI** |
| Intercept | 0.16 | | 0.50 | | -0.82 | | 1.16 |
| SMI_norm | -0.53 | | 0.22 | | -0.99 | | -0.11 |
| TelomerLength_norm | -0.19 | | 0.27 | | -0.72 | | 0.32 |
|  |  | |  | |  | |  |
| Bank voles: number of ticks ~ SMI_norm + TelomerLength_norm + (1 \| plotID) | | | | | | | |
|  | **Estimate** | | **Est.Error** | | **l-95% CI** | | **u-95% CI** |
| Intercept | -0.25 | | 0.26 | | -0.85 | | 0.23 |
| SMI_norm | -0.09 | | 0.09 | | -0.28 | | 0.09 |
| TelomerLength_norm | -0.09 | | 0.11 | | -0.31 | | 0.12 |
|  |  | |  | |  | |  |
| Wood mice: pathogen_presence ~ SMI_norm + TelomerLength_norm + (1 \| plotID) | | | | | | | |
|  | **Estimate** | | **Est.Error** | | **l-95% CI** | | **u-95% CI** |
| Intercept | -1.58 | | 0.32 | | -2.26 | | -1.01 |
| SMI_norm | -0.07 | | 0.27 | | -0.63 | | 0.46 |
| TelomerLength_norm | -0.20 | | 0.27 | | -0.73 | | 0.32 |
|  |  | |  | |  | |  |
| Wood mice: Spiroplasma ~ SMI_norm + TelomerLength_norm + (1 \| plotID) | | | | | | | |
|  | **Estimate** | | **Est.Error** | | **l-95% CI** | | **u-95% CI** |
| Intercept | -3.18 | | 0.67 | | -4.91 | | -2.18 |
| SMI_norm | 0.24 | | 0.44 | | -0.61 | | 1.11 |
| TelomerLength_norm | 0.20 | | 0.50 | | -0.68 | | 1.31 |
|  |  | |  | |  | |  |
| Wood mice: R.helvetica ~ SMI_norm + TelomerLength_norm + (1 \| plotID) | | | | | | | |
|  | **Estimate** | | **Est.Error** | | **l-95% CI** | | **u-95% CI** |
| Intercept | -2.82 | | 0.59 | | -4.18 | | -1.86 |
| SMI_norm | -0.44 | | 0.42 | | -1.34 | | 0.33 |
| TelomerLength_norm | -0.18 | | 0.39 | | -0.93 | | 0.59 |
|  |  | |  | |  | |  |
| Wood mice: B. burgdorferi s.l. ~ SMI_norm + TelomerLength_norm + (1 \| plotID) | | | | | | | |
|  | **Estimate** | | **Est.Error** | | **l-95% CI** | | **u-95% CI** |
| Intercept | -3.77 | | 0.86 | | -5.86 | | -2.45 |
| SMI_norm | 0.10 | | 0.50 | | -0.91 | | 1.07 |
| TelomerLength_norm | -0.88 | | 0.58 | | -2.11 | | 0.17 |
|  |  | |  | |  | |  |
| Wood mice: tick presence ~ SMI_norm + TelomerLength_norm + (1 \| plotID) | | | | | | | |
|  | **Estimate** | | **Est.Error** | | **l-95% CI** | | **u-95% CI** |
| Intercept | 2.44 | | 0.63 | | 1.44 | | 3.98 |
| SMI_norm | -0.62 | | 0.34 | | -1.27 | | 0.05 |
| TelomerLength_norm | 0.56 | | 0.39 | | -1.33 | | 0.20 |
|  |  | |  | |  | |  |
| Wood mice: number of ticks ~ SMI_norm + TelomerLength_norm + (1 \| plotID) | | | | | | | |
|  | **Estimate** | | **Est.Error** | | **l-95% CI** | | **u-95% CI** |
| Intercept | 1.27 | | 0.20 | | 0.87 | | 1.66 |
| SMI_norm | 0.25 | | 0.05 | | 0.15 | | 0.35 |
| TelomerLength_norm | 0.19 | | 0.07 | | 0.06 | | 0.32 |

**References**

Callicott, R.J., Womack, J.E., 2006. Comparative Medicine Real-time PCR Assay for Measurement of Mouse Telomeres.

Cawthon, R.M., 2002. Telomere measurement by quantitative PCR, Nucleic Acids Research.

Marshall, P.L., Davis, G., Taylor, S.W., 2003. Using Line Intersect Sampling for Coarse Woody Debris: Practitioners Questions Addressed, Forest Research technical report. Nanaioma, British Comombia, Canada.
